# Effect of a brief intervention for alcohol and illicit drug use on trauma recidivism in a cohort of trauma patients

**DOI:** 10.1371/journal.pone.0182441

**Published:** 2017-08-16

**Authors:** Sergio Cordovilla-Guardia, Enrique Fernández-Mondéjar, Raquel Vilar-López, Juan F. Navas, Mónica Portillo-Santamaría, Sergio Rico-Martín, Pablo Lardelli-Claret

**Affiliations:** 1 Nursing Department, Nursing and Occupational Therapy College, University of Extremadura, Cáceres, Spain; 2 Servicio de Medicina Intensiva, Complejo Hospitalario Universitario de Granada, Granada, Spain; 3 Instituto de Investigación Biosanitaria IBS, Granada, Spain; 4 Department of Personality, Evaluation and Psychological Treatment. University of Granada, Granada, Spain; 5 Addictive Disorders Network, RTA Instituto de Salud Carlos III, Spanish Ministry, Spain; 6 Mind, Brain and Behavior Research Centre, University of Granada, Granada, Spain; 7 Department of Experimental Psychology. University of Granada, Granada, Spain; 8 Servicio de Salud Mental, Hospital de la Ribera, Valencia, Spain; 9 Department of Preventive Medicine and Public Health, School of Medicine, University of Granada, Granada, Spain. CIBER of Epidemiology and Public Health. Spain; Pennsylvania State University College of Medicine, UNITED STATES

## Abstract

**Objective:**

Estimate the effectiveness of brief interventions in reducing trauma recidivism in hospitalized trauma patients who screened positive for alcohol and/or illicit drug use.

**Methods:**

Dynamic cohort study based on registry data from 1818 patients included in a screening and brief intervention program for alcohol and illicit drug use for hospitalized trauma patients. Three subcohorts emerged from the data analysis: patients who screened negative, those who screened positive and were offered brief intervention, and those who screened positive and were not offered brief intervention. Follow-up lasted from 10 to 52 months. Trauma-free survival, adjusted hazard rate ratios (aHRR) and adjusted incidence rate ratios (aIRR) were calculated, and complier average causal effect (CACE) analysis was used.

**Results:**

We found a higher cumulative risk of trauma recidivism in the subcohort who screened positive. In this subcohort, an aHRR of 0.63 (95% CI: 0.41–0.95) was obtained for the group offered brief intervention compared to the group not offered intervention. CACE analysis yielded an estimated 52% reduction in trauma recidivism associated with the brief intervention.

**Conclusion:**

The brief intervention offered during hospitalization in trauma patients positive for alcohol and/or illicit drug use can halve the incidence of trauma recidivism.

## Introduction

Traumatic injury related to alcohol and illicit drug use remains an important public health challenge [1,2]. Among other health problems, the use of these substances is frequently associated with trauma recidivism [3–7]. The use of screening, brief intervention, and referral to treatment (SBIRT) programs in trauma centers [8] is spreading as an evidence-based measure which may enhance the impact of preventive efforts in this population [9]. Brief intervention (BI) is a counseling approach based on the principles of motivational interviewing [10], a collaborative person-centered form of guidance intended to elicit and strengthen motivation for change [11]. Brief interventions usually consist of one to four individual interviews lasting 7.5 to 60 minutes each [12,13], and are usually conducted by psychologists, nurses or doctors with specific training [12], although they can be successfully implemented by other health care practitioners [14]. Considerable evidence documents the usefulness of BI to combat problematic alcohol use in primary care [12], general hospitals [13] and trauma centers [13,15]. Admission to trauma centers offers a potential “teachable moment” because patients may have perceptions of vulnerability about their health and therefore may be particularly receptive to screening and counseling [16]. Numerous studies have reported the short-term effectiveness of a single BI session in reducing alcohol consumption when the session takes place during admission in these clinical settings [15,17–19], especially when BI is combined with a telephone booster after discharge [15,17,18,20,21]. However, the results were relatively modest after 12 months of follow-up. There is also evidence of the effectiveness of BI in reducing illegal drug consumption [22] or both alcohol and illicit drug use [23].

In light of this evidence, the American College of Surgeons passed a resolution in 2005 requiring level I trauma centers in the USA to have a mechanism for screening injured patients for alcohol-use disorder and providing an intervention to patients who screen positive [24]. This mandate greatly increased the dissemination of SBIRT programs [25]. In 2011, encouraged by this expansion, we implemented a project based on SBIRT (the MOTIVA project) targeted for patients hospitalized at our center for trauma related to alcohol and illicit drug use [26].

Unfortunately, the well-documented effect of BI in reducing alcohol or illicit drug consumption has not been accompanied by similar evidence of reductions in health events theoretically related to substance use. Thus far, the effect of BI on injury recurrence remains unclear. Gentilello et al. [27] tested the effect of brief alcohol intervention in a trauma center to reduce recidivisms. They found a 47% reduction in injuries requiring emergency department care or trauma center admission during the first year. An almost identical reduction in inpatient hospital readmissions (48%) was found in patients in the intervention group with up to 3 years of follow-up. However, neither of these estimates reached statistical significance. A similar reduction (41%) was found in a later metaanalysis [28] that combined the results from three studies [20,29,30] which considered trauma recidivism as a secondary outcome. However, the heterogeneity between studies regarding major design factors such as age of participants (adolescents 13 to 17 years old [29], older adolescents aged 18–19 years [30] or with no reported age range [20]) and length of follow-up (6 months [30] or 12 months [20,29]) raise questions concerning the validity of this estimate. More recently, Woolard et al., (2013) [31] evaluated the effect of BI for patients seen in the emergency department for alcohol and marijuana use after 12 months of follow-up. Although they found a decrease in binge drinking and conjoint use, the anticipated reductions in injury rates were not found.

Undoubtedly, methodological drawbacks shared by most previous studies [20,29–31] make it hard to offer valid evidence of the effectiveness of BI. For example, short follow-up periods (no more than 12 months) provide no information on the longer-term effects of BI, and the self-reported nature of the main outcome (recidivism) raises the possibility of differential misclassification bias.

In an effort to overcome these limitations we designed the present study to evaluate the effectiveness of BI in patients hospitalized for trauma who screened positive for alcohol and/or illicit drug use. Our main objective was to study the effect of BI on reductions in trauma recidivism after 10 to 52 months of follow-up. This research was accordingly designed to test two hypotheses:

- The recidivism rate in patients who screened negative for alcohol or illicit drug use is lower than in patients who tested positive and did not receive BI.
- In the subgroup of patients who screened positive for alcohol or illicit drug use, the recidivism rate in those who receive BI is lower than in patients who did not receive BI.

## Methods

### The MOTIVA project

This retrospective, region-wide, dynamic cohort study was based on data obtained from the MOTIVA project, with passive and active follow-up lasting from 10 to 52 months and was approved by the Granada Provincial Research Ethics Committee (CEI-Granada).

**Fig 1:**
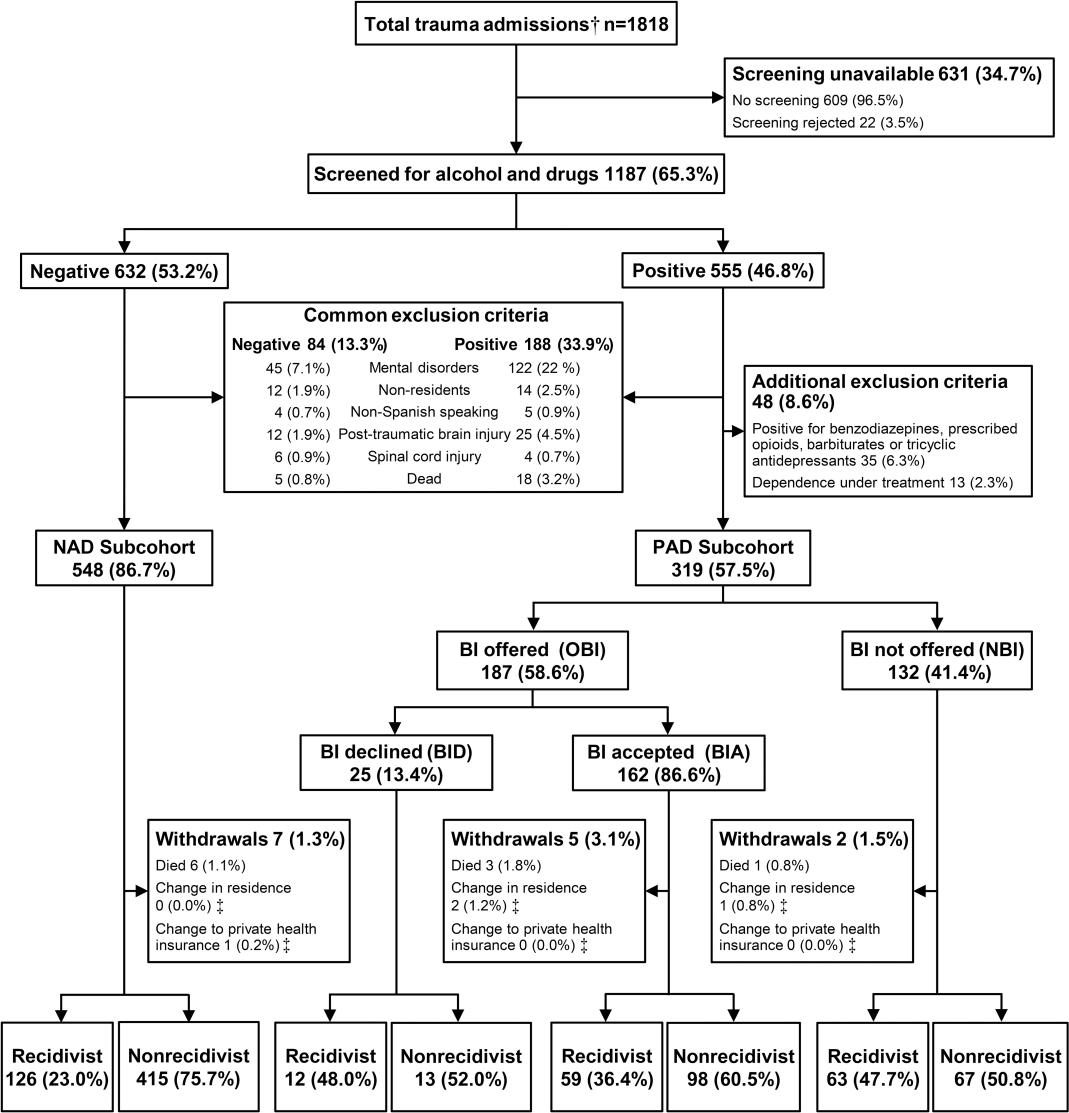
Flowchart of the distribution of patients. † Patients between 16 and 70 years old admitted. ‡ Active follow-up by telephone interview (NAD: n = 91, BI accepted: n = 151, BI offered: n = 113). NAD: Negative for alcohol and/or drugs. PAD: Positive for alcohol and/or drugs. BI: Brief Intervention. No withdrawals in BI rejected group.

The MOTIVA project was a SBIRT-based program implemented in November 2011 at University Hospital of Granada (UHG). This center is a public tertiary-care hospital located in Andalusia, an autonomous region in southern Spain, and as part of the public health national system it covers a population of more than 600,000 inhabitants. The MOTIVA project was active during the 31 nonconsecutive months during which it received financial support from the Regional Andalusian Government and the Spanish National Traffic Directorate: November 2011 to October 2012, June 2013 to November 2013, and June 2014 to June 2015. The reference population for this project was all patients aged 16 to 70 years who were hospitalized for traumatic injuries. The MOTIVA project comprised the following activities:

a) Screening for alcohol and drugs. Of all 1818 patients aged 16 to 70 years who were hospitalized for trauma during the study periods (Fig 1), 1187 (65.3%) could be screened for alcohol and drug use; 609 patients were not screened and 22 refused screening. Informed consent was requested for alcohol and drug testing. In sedated patients or unable to collaborate, samples were collected at admission and consent to access the results of the screening was solicited when the patient´s clinical situation was resolved. When this was not possible, the consent was requested to the relatives. Alcohol consumption was screened by blood testing, and was considered positive when the blood alcohol level was higher than 0.3 g/L. For patients from whom a blood sample could not be obtained, the Alcohol Use Disorders Identification Test (AUDIT-C) was used, and the result was considered positive for patients who were admitted for problem drinking [32]. An AUDIT-C score of 4 or more in men and 3 or more in women was considered positive. Screening for other drugs (cannabis, cocaine, amphetamines, methamphetamines, benzodiazepines, opiates, methadone, barbiturates or tricyclic antidepressants) was done with urine testing by fluorescence immunoassay. Reviews of the patients’ medical records were used to rule out patients who tested positive for benzodiazepines and opioids as a result of emergency treatment of their injury. Overall, 555 patients (46.8% of those screened) tested positive for alcohol or drugs. For the purpose of this study, we excluded from the cohort screened patients who met the following exclusion criteria: nonresidents in Andalusia, non-Spanish speaking, post-traumatic brain injury, mental disorders, spinal cord injury, and death during hospital stay. Two additional exclusion criteria were used for positive patients: positive screening result due to prescribed use of benzodiazepines, opioids, barbiturates or tricyclic antidepressants, and drug dependence under treatment. Therefore the final cohort comprised 867 patients, classified in two subcohorts: negative for alcohol and drugs (NAD: 548 patients) and positive for alcohol and/or drugs (PAD: 319 patients).

b) Brief intervention. Patients included in the PAD subcohort were candidates to receive BI. The convalescence period just before hospital discharge was considered the best window of opportunity for the interview, but some patients were discharged without receiving the BI, mainly those with a short hospital stay. Therefore BI was not offered to 132 (41.4%) eligible patients (NBI). In the remaining group of 187 for whom BI was offered (OBI), 25 (13.4%) patients who agreed to be screened declined the BI (BID group). Therefore a final total of 162 patients accepted and received the BI (BIA group).

The BI consisted of an interview (30 to 45 min) based on motivational interviewing principles [10]. All interviews consisted of six components.

1. Introduction starting with communication of the screening results and explanation of the aim of the intervention. We sought a positive response indicating the patient’s willingness to participate in the intervention, through the expression of interest and concern with an empathic therapeutic approach and efforts to encourage confidence.
2. Exploration of the motivation for consumption and review of potential negative consequences, to favor discovery of the pros and cons of current substance use.
3. Personalized normative feedback about the patient’s pattern of alcohol/drug use and risks, and resolution of ambivalence with nonconfrontational responses to resistance.
4. Discussion of possible future situations that might arise from the patient’s current consumption of alcohol and/or drugs versus a change in consumption behavior.
5. When the level of motivation to change allowed: negotiation of consumption goals, identifying and anticipating potential barriers and establishing strategies to overcome them, favoring self-efficacy.
6. Final summary in which the patient was asked to state his or her conclusions, and any remaining questions were answered.

In all cases the patients were informed about community resources for problems with alcohol and illicit drug use. Patients were contacted by telephone 3 months after hospital discharge for a 10–15 min booster session to increase motivation to pursue their goals. The telephone booster session was performed in 123 (75.9%) patients of the BIA group. All interventions were conducted by a nurse or psychologist with the same specific training in BI. This training consisted of instruction, demonstrations and active learning exercises [33] provided by a clinical psychologist with extensive experience in motivational interviewing.

c) Registry of participants. A specially designed registry of all screened patients included information about the screening test results and BI implementation. The following additional information obtained prospectively from the medical record during the hospital stay was also included the registry: age, sex, mechanism of injury, Injury Severity Score (ISS) [34], diagnosis of psychiatric comorbidity, days of hospitalization and hospital mortality.

In order to complete the information regarding past trauma history (PTHx) of the patients included in the study, the Andalusian Regional Health Service Database (Diraya®) [35] was also consulted. This database, in operation since 1999, includes the patients’ medical history and records of any health care received at more than 1500 centers operated by the Andalusian Public Health Service.

### Follow-up

The three resulting subcohorts (548 NAD patients, 132 NBI patients and 187 OBI patients) were followed up with two procedures: passive follow-up and active follow-up.

#### Passive follow-up

During the period from March to June 2016 the digital medical records (from the Diraya® database) of patients from all three subcohorts were reviewed to search for trauma recidivism up until March 1, 2016. Trauma recidivism was defined as the occurrence of a new traumatic injury requiring medical care at any center belonging to the regional public health system. The nurses who conducted this review were blinded to exposure status, and collected information on the occurrence of a new trauma, date, injury mechanism and ISS. To detect deaths during follow-up, those that occurred in any health care facility were also searched for in the Diraya® database. In addition, we consulted the database of the Provincial Institute of Forensic Medicine and funeral service records for the same period.

#### Active follow-up

For purposes of comparison with passive follow-up data, active telephone follow-up was used for all PAD patients and a random sample of 91 NAD patients. To estimate the sample size we assumed an expected recidivism of 22% [36] and loss to follow-up less than 5%. In the telephone interview we ask each patient about the same variables as were used in passive follow-up, plus information about withdrawal from the cohort due to change of residence to another region outside Andalusia (2 in the BIA group and 1 in the NBI group) or change to private health insurance (1 patient in the NAD group).

### Definition of study variables

For exposure we defined three main subgroups of patients: NAD, OBI and NBI. Patients who screened as PAD were subclassified into the following categories: consumers of alcohol (only alcohol detected), cannabis (only cannabis), cocaine-amphetamine (positive for amphetamine, methamphetamine and/or cocaine) and polydrugs (including any combination of two or more of the above groups). Exposure to heroin and methadone, when detected, was always accompanied by exposure to at least one other substance in this study, so all patients who screened positive for these two drugs were included in the polydrugs group. Patients in the OBI subcohort were classified according to whether they accepted (BIA group) or declined BI (BID group).

Cohen’s kappa index was used to estimate concordance between the percentages of recidivists (any new trauma) found by active and passive follow-up method. On the basis of the passive follow-up, we defined two outcome variables for each patient:

- Number of traumatic injuries during follow-up. This variable allowed us to estimate the incidence rate of trauma in each subcohort.
- Time from hospital discharge up to the first new trauma, withdrawal or the end of follow-up with no new injury (March 1, 2016).

As potential confounders we recorded the following at baseline: age, length of hospital stay (continuous), sex (male or female), mechanism of injury (traffic, sports, assault, falls on the same level, falls from a height, cuts or bruises, and other mechanisms), injury severity categorized into three levels according to ISS (mild: 1 to 8, moderate: 9 to 15, and severe: ≥16), and PTHx classified into three levels (nonrecidivist: first-time trauma patient, single recidivist: only one previous trauma, and multirecidivist: patients with more than one previous trauma).

### Analysis

A descriptive analysis is reported here of the patients’ baseline characteristics and outcomes in each subcohort. To evaluate the effect of BI on trauma recidivisms, two complementary strategies were used: intention-to-treat (ITT) analysis (comparison of the OBI and NBI groups) and per-protocol (PP) analysis (comparison of BIA and NBI groups). The Kaplan–Meier product-limit method and the log-rank test were used to estimate and compare curves for survival without new trauma events in each subcohort. Cox proportional hazards regression was used to obtain adjusted hazard rate ratios (HRR) to estimate the strength of association between each exposure level and the incidence of first traumatic events, including all baseline characteristics as covariates. For the total number of trauma events during the entire follow-up period for each patient as the dependent variable, a Poisson regression model was used to obtain the corresponding adjusted incidence rate ratios (IRR). Likelihood ratio tests (lrtest) were used to examine the potential statistical interaction between BI and PTHx.

Additionally, to compare the OBI and NBI groups, complier average causal effect (CACE) analysis [37] was used to obtain adjusted IRR estimates in the hypothetical subgroup of patients who would have agreed to receive the intervention if it had been offered.

All data analyses were done with Stata Statistical Software, Release 14 (StataCorp. 2015, College Station, TX, USA).

## Results

Of the 1187 patients screened, 555 (46.8%) were positive. After the exclusion criteria for BI were applied, we obtained a cohort of 867 patients (548 NAD patients, 319 PAD patients). Differences in the baseline characteristics between groups (Table 1) were observed for age (higher median age in the NAD group), sex (higher proportion of females in the NAD group) and PHTx (much higher frequency of nonrecidivism in the NAD group). When we compared the NBI and OBI groups, the main difference, as expected, was in length of hospital stay, which was much longer in the OBI group. The proportions of mild injuries and polydrug use were lower in the OBI group, whereas cannabis use was more frequent.

**Table 1:** Baseline characteristics of the groups.

		PAD	
	NAD (n = 548)	OBI (n = 187)	NBI (n = 132)
**Age (years)** Median [IQR]	43 [30–55]	36 [26–49]	38 [26–51]
**Sex** n (%)			
Male	369 (67.3)	153 (81.8)	113 (85.6)
**Mechanism of injury** n (%)			
Traffic collision	157 (28.6)	58 (31.0)	30 (22.7)
Sports injury	60 (10.9)	20 (10.7)	3 (2.3)
Assault	10 (1.8)	19 (10.2)	18 (13.6)
Falls on the same level	169 (30.8)	46 (24.6)	40 (30.3)
Falls from a height	63 (11.5)	21 (11.2)	21 (15.9)
Cuts or bruises	65 (11.9)	13 (7.0)	17 (12.9)
Other mechanisms	24 (4.4)	10 (5.3)	3 (2.3)
**Injury Severity Score** n (%)			
Mild: 1 to 8	419 (76.5)	130 (69.5)	101 (76.5)
Moderate: 9 to 15	97 (17.7)	43 (23.0)	20 (15.2)
Severe: ≥16	32 (5.8)	14 (7.5)	11 (8.3)
**Days of hospitalization** Median [IQR]	4 [3–8]	6 [4–11]	1 [1–2]
**Substance detected** n (%)			
Alcohol	—	71 (38.0)	50 (37.9)
Cannabis	—	34 (18.3)	12 (9.1)
Cocaine-amphetamine	—	8 (4.3)	8 (6.1)
Polydrugs	—	74 (39.6)	62 (47.0)
**Past trauma history n (%)**			
Nonrecidivist	291 (53.1)	49 (30.2)	42 (31.8)
Single recidivist	164 (29.9)	57 (35.2)	41 (31.1)
Multirecidivist	93 (17.0)	56 (34.6)	49 (37.1)

NAD: Negative for alcohol and/or drugs. PAD: Positive for alcohol and/or drugs. OBI: Offered brief intervention group. NBI: Not offered brief intervention group. IQR: Interquartile range. Alcohol: Positive only for alcohol. Cannabis: Positive only for cannabis. Cocaine-amphetamine: Positive only for cocaine, amphetamines and/or methamphetamines. Polydrugs: Positive for any combination of substances in the above groups and nonprescribed opiates. Nonrecidivist: First-time trauma patients. Single recidivist: Patients with only one previous trauma. Multirecidivist: Patients with more than one previous trauma.

The concordance for recidivism between passive and active follow-up was high (Table 2). However, active follow-up detected a lower number of new injuries in the OBI group (55 vs. 62 with passive follow-up).

**Table 2:** Analysis of concordance between passive and active follow-up of first trauma.

			PAD			
	NAD (n = 91)†		OBI (n = 170)†		NBI (n = 113)†	
	Passive	Active	Passive	Active	Passive	Active
**Trauma recidivist** n (%)	24 (26.4)	27 (29.7)	62 (36.5)	55 (32.4)	55 (48.7)	52 (46.0)
Kappa (95% CI)	0.92 (0.83–1.00)		0.86 (0.77–0.94)		0.95 (0.89–1.00)	
*p* value	<0.001		<0.001		<0.001	
	**(n = 24)**‡		**(n = 53)**‡		**(n = 52)**‡	
**Mechanism of injury** n (%)						
Traffic collision	5 (20.8)	5 (20.8)	17 (32.1)	16 (30.2)	13 (25.0)	12 (23.1)
Sports injury	1 (4.2)	1 (4.2)	0 (0.0)	1 (1.9)	3 (5.8)	3 (5.8)
Assault	1 (4.2)	1 (4.2)	6 (11.3)	9 (17.0)	7 (13.5)	10 (19.2)
Falls on the same level	11 (45.8)	11 (45.8)	18 (34.0)	17 (32.1)	13 (25.0)	13 (25.0)
Falls from a height	0 (0.0)	0 (0.0)	2 (3.8)	3 (5.7)	1 (1.9)	1 (1.9)
Cuts or bruises	6 (25.0)	6 (25.0)	9 (17.0)	6 (11.3)	12 (23.1)	12 (23.1)
Other mechanisms	0 (0.0)	0 (0.0)	1 (1.9)	1 (1.9)	3 (5.8)	1 (1.9)
Kappa (95% CI)	1.00 (1.00–1.00)		0.85 (0.74–0.96)		0.83 (0.72–0.94)	
*p value*	*<0*.*001*		*<0*.*001*		*<0*.*001*	
**Injury Severity Score** n (%)						
Mild: 1 to 8	24 (100)	24 (100)	50 (94.3)	51 (95.5)	47 (90.4)	47 (90.4)
Moderate: 9 to 15	0 (0.0)	0 (0.0)	3 (2.3)	2 (4.5)	3 (5.8)	3 (5.8)
Severe: ≥16	0 (0.0)	0 (0.0)	0 (0.0)	0 (0.0)	2 (3.8)	2 (3.8)
Kappa (95%CI)	1.00 (1.00–1.00)		0.64 (0.19–1.00)		0.78 (0.49–1.00)	
*p* value	<0.001		<0.001		<0.001	

† Patients with data from both follow-up methods.

‡ Recidivist patients with data from both follow-up methods. Passive: Follow-up by Diraya® health information system. Active: Follow-up by telephone interview. NAD: Negative for alcohol and/or drugs. PAD: Positive for alcohol and/or drugs. OBI: Offered brief intervention group. NBI: Not offered brief intervention group. CI: Confidence interval.

According to data from passive follow-up, the incidence rate of trauma recidivism was 8.7 per 100 patient-years in the NAD subcohort, 14.1 per 100 patient-years in the OBI subcohort, 13.0 per 100 patient-years in the BIA subcohort and 25.4 per 100 patient-years in the NBI subcohort. Subsequent trauma after discharge took place a median of 16 months earlier in the NBI group than in the OBI group (Table 3); however, there were no significant differences between groups in the mechanism, severity of injury or percentage of hospitalized trauma patients.

**Table 3:** Characteristics of the first new trauma in each patient during follow-up†.

		PAD		
	NAD (n = 126)	OBI (n = 71)	NBI (n = 63)	OBI vs. NBI *p* value
**Months follow-up to first trauma** Median [IQR]	32 [17–48]	33 [17–49]	17 [11–34]	0.001^A^
**Mechanism of injury** n (%)				
Traffic collision	22 (17.5)	22 (31.0)	15 (23.8)	0.319^B^
Sports injury	8 (6.3)	0 (0.0)	4 (6.3)	
Assault	4 (3.2)	7 (9.9)	8 (12.7)	
Falls on the same level	57 (45.2)	21 (29.6)	16 (25.4)	
Falls from a height	4 (3.2)	3 (4.2)	1 (1.6)	
Cuts or bruises	26 (20.6)	15 (21.1)	14 (22.2)	
Other mechanisms	5 (4.0)	3 (4.2)	5 (7.9)	
**Injury Severity Score** n (%)				
Mild: 1 to 8	125 (99.2)	67 (94.4)	55 (93.7)	0.358^B^
Moderate: 9 to 15	1 (0.8)	3 (4.2)	5 (6.3)	
Severe: ≥16	0 (0.0)	1 (1.7)	3 (3.2)	
**Hospitalized** n (%)	29 (23.0)	18 (25.3)	19 (30.1)	0.534^A^

† Passive follow-up by Diraya® health information system. NAD: Negative for alcohol and/or drugs. PAD: Positive for alcohol and/or drugs. OBI: Offered brief intervention group. NBI: Not offered brief intervention group. IQR: Interquartile range.

^A^Mann–Whitney test.

^B^Chi-squared exact test.

**Fig 2:**
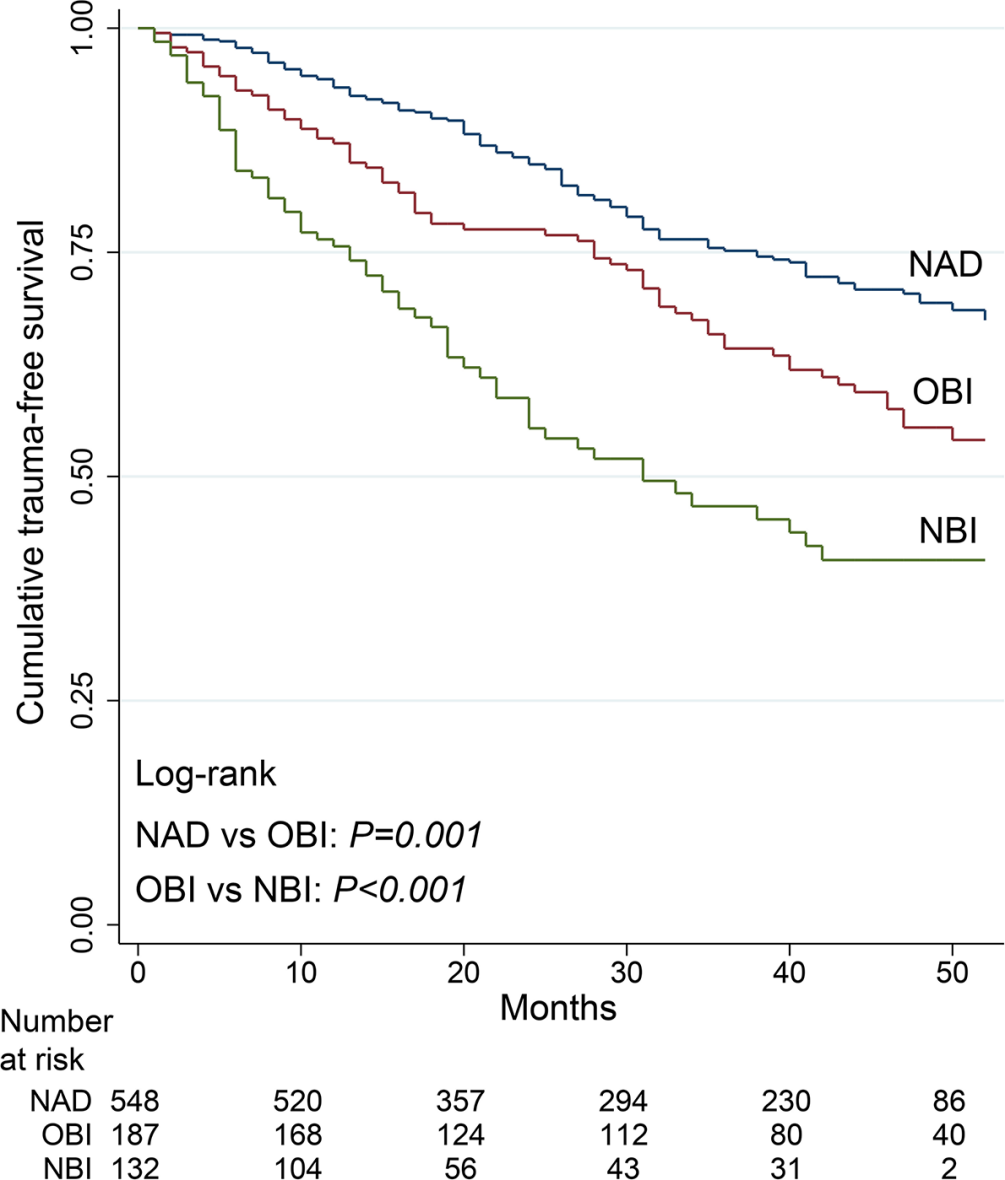
Kaplan–Meier curves of trauma-free survival in follow-up patients. NAD: Negative for alcohol and/or drugs. OBI: Offered brief intervention group. NBI: Not offered brief intervention group.

**Fig 3:**
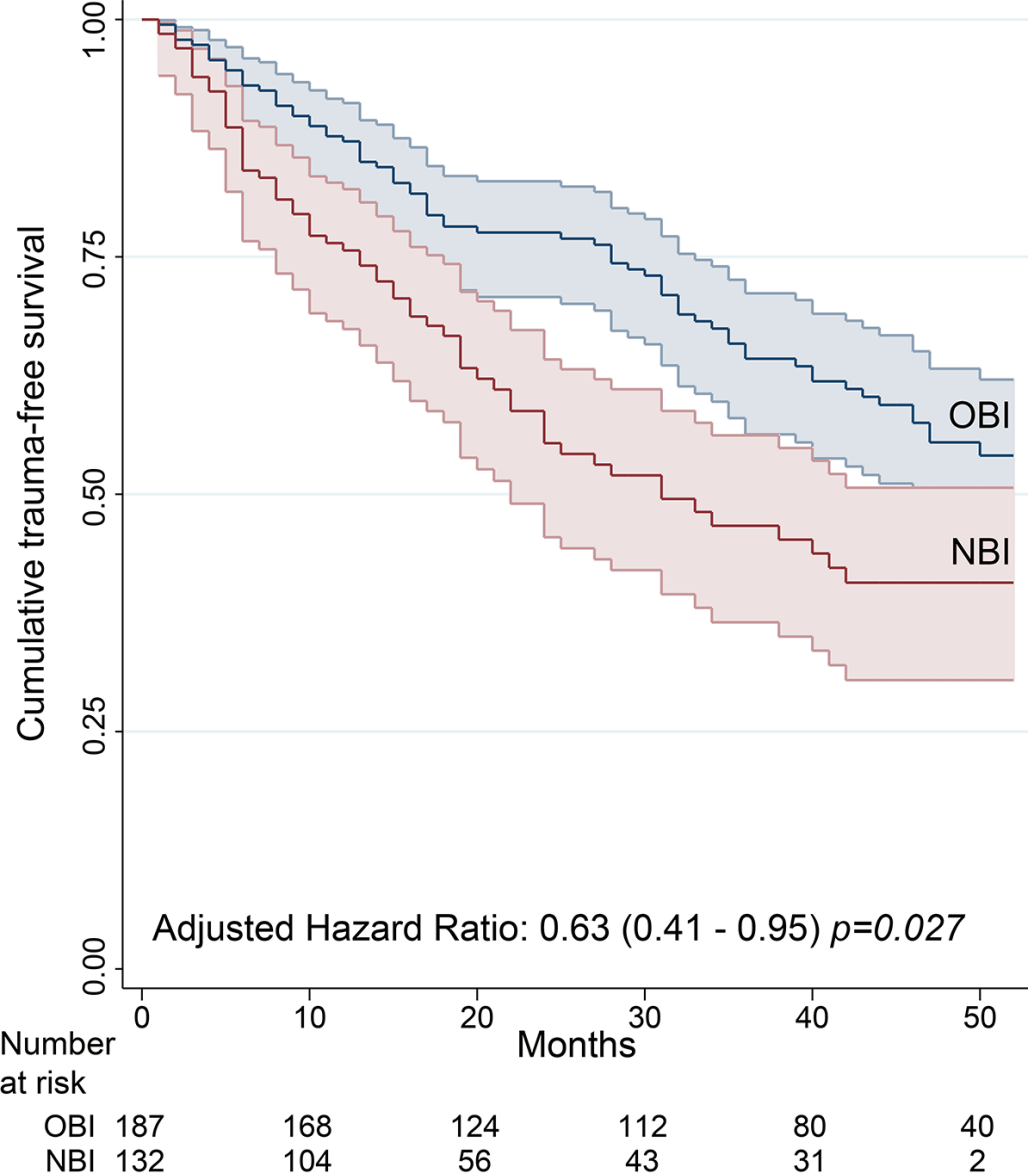
Kaplan–Meier curves of trauma-free survival in follow-up patients positive for substances. Hall–Wellner bands represent 95% confidence intervals. OBI: Offered brief intervention group. NBI: Not offered brief intervention group. Adjusted hazard ratio using Cox proportional hazards regression model with covariables age, sex, mechanism of injury, Injury Severity Score, days of hospitalization, substance detected and past trauma history. NBI as the reference group.

Kaplan–Meier curves (Fig 2) showed a significantly greater cumulative risk of recidivism in the two PAD subcohorts (OBI and NBI) compared to the NAD group. In the PAD subcohort, longer trauma-free survival was observed for the OBI group than the NBI group (Fig 3).

The results of multivariate regression analysis for the entire cohort are shown in Table 4. According to the Cox proportional model with the NAD group as the reference, the adjusted HRR was 1.31 (95% CI: 0.96–1.78) for the OBI group and 2.14 (95% CI: 1.53–2.98) for the NBI group. Other variables related with recidivism were age (inversely associated) and PTHx (positively associated). The corresponding values for adjusted IRR were similar. There was no evidence of interaction between BI and PTHx on the risk of trauma recidivism (*p* = 0.754 in the lrtest).

**Table 4:** Multivariate regression models for the entire cohort (NAD + PAD).

	Cox proportional model aHRR (95%CI)	Poisson model aIRR (95%CI)
**Exposure**		
NAD	1.00 Ref.	1.00 Ref.
OBI	1.31 (0.96–1.78)	1.24 (0.95–1.62)
NBI	2.14 (1.53–2.98)	2.15 (1.63–2.83)
**Age**		
1-year increase	0.98 (0.97–0.99)	0.98 (0.97–0.99)
**Sex**		
Female	1.00 Ref.	1.00 Ref.
Male	1.12 (0.81–1.55)	1.08 (0.82–1.43)
**Injury Severity Score**		
Mild: 1 to 8	1.00 Ref.	1.00 Ref.
Moderate: 9 to 15	0.90 (0.61–1.31)	0.87 (0.63–1.22)
Severe: ≥16	1.12 (0.64–1.96)	0.85 (0.51–1.42)
**Days of hospitalization**		
1-day increase	0.99 (0.97–1.01)	0.99 (0.97–1.01)
**Mechanism of injury**		
Traffic collision	1.00 Ref.	1.00 Ref.
Sports injury	0.66 (0.39–1.10)	0.70 (0.44–1.11)
Assault	1.12 (0.69–1.81)	1.03 (0.68–1.55)
Falls on the same level	1.27 (0.89–1.81)	1.38 (1.02–1.87)
Falls from a height	1.06 (0.67–1.68)	1.06 (0.72–1.56)
Cuts or bruises	1.12 (0.73–1.71)	1.14 (0.80–1.62)
Other mechanisms	0.46 (0.19–1.16)	0.44 (0.17–1.08)
**Past trauma history**		
Nonrecidivist	1.00 Ref.	1.00 Ref.
Single recidivist	1.53 (1.12–2.11)	1.67 (1.27–2.19)
Multirecidivist	2.59 (1.90–3.54)	2.45 (1.86–3.21)

aHRR: Adjusted hazard rate ratio. aIRR: Adjusted incidence rate ratio using Poisson regression. NAD: Negative for alcohol and/or drugs. PAD: Positive for alcohol and/or drugs. OBI: Offered brief intervention group. NBI: Not offered brief intervention group. Nonrecidivist: First-time trauma patients. Single recidivist: Patients with only one previous trauma. Multirecidivist: Patients with more than one previous trauma.

When the analysis was restricted to PAD patients (Table 5) the adjusted HRR was 0.63 (95% CI: 0.41–0.95) for the OBI group compared to the NBI group. This lower risk of recidivism increased to 0.55 (95% CI: 0.36–0.85) in the per-protocol analysis (i.e., when we compared the NBI and BIA groups). The corresponding adjusted IRR values were 0.61 (95% CI: 0.43–0.86) for the OBI group and 0.45 (95% CI: 0.3–0.66) for the BIA group. The CACE analysis yielded an adjusted IRR of 0.48 (95% CI: 0.24–0.98). The pattern of association for the remaining baseline variables was not substantially different from that obtained for the entire cohort; only lower age and PTHx were significantly associated with trauma recidivism.

**Table 5:** Multivariate regression models for the PAD subcohort.

	Cox proportional model aHRR (95%CI)		Poisson model aIRR (95%CI)		
	ITT	PP	ITT	PP	CACE
**Exposure**					
NBI	1.00 Ref.	1.00 Ref.	1.00 Ref.	1.00 Ref.	1.00 Ref.
OBI	0.63 (0.41–0.95)	—	0.61 (0.43–0.86)	—	0.48 (0.24–0.98)
BIA	—	0.55 (0.36–0.85)	—	0.45 (0.31–0.66)	—
**Age**					
1-year increase	0.98 (0.96–0.99)	0.98 (0.96–0.99)	0.98 (0.97–0.99)	0.98 (0.97–0.99)	0.97 (0.94–0.98)
**Sex**					
Female	1.00 Ref.	1.00 Ref.	1.00 Ref.	1.00 Ref.	1.00 Ref.
Male	1.14 (0.65–1.98)	1.01 (0.58–1.77)	1.19 (0.76–1.87)	1.01 (0.63–1.60)	1.64 (0.82–3.30)
**Injury Severity Score**					
Mild: 1 to 8	1.00 Ref.	1.00 Ref.	1.00 Ref.	1.00 Ref.	1.00 Ref.
Moderate: 9 to 15	1.05 (0.65–1.73)	0.92 (0.55–1.57)	1.03 (0.67–1.59)	0.95 (0.60–1.50)	1.13 (0.66–1.92)
Severe: ≥16	1.34 (0.69–2.59)	1.43 (0.73–2.79)	0.95 (0.53–1.70)	1.01 (0.56–1.83)	0.76 (0.39–1.52)
**Days of hospitalization**					
1-day increase	0.98 (0.96–1.01)	0.99 (0.96–1.02)	0.98 (0.95–1.01)	0.98 (0.95–1.02)	0.99 (0.92–1.05)
**Mechanism of injury**					
Traffic collision	1.00 Ref.	1.00 Ref.	1.00 Ref.	1.00 Ref.	1.00 Ref.
Sports injury	0.59 (0.27–1.28)	0.60 (0.27–1.33)	0.76 (0.38–1.51)	0.86 (0.42–1.72)	1.30 (0.58–2.89)
Assault	0.89 (0.51–1.56)	0.84 (0.47–1.49)	0.88 (0.55–1.42)	0.85 (0.52–1.38)	1.26 (0.63–2.50)
Falls on the same level	0.86 (0.50–1.47)	0.68 (0.38–1.22)	1.23 (0.78–1.91)	0.93 (0.56–1.55)	2.14 (1.00–4.60)
Falls from a height	1.07 (0.59–1.94)	1.06 (0.58–1.95)	1.19 (0.78–1.95)	1.13 (0.68–1.86)	1.12 (0.62–2.03)
Cuts or bruises	0.78 (0.41–1.49)	0.59 (0.29–1.19)	1.07 (0.66–1.75)	0.65 (0.36–1.16)	1.16 (0.58–2.30)
Other mechanisms	0.33 (0.09–1.40)	0.34 (0.08–1.47)	0.32 (0.08–1.34)	0.35 (0.08–1.47)	0.36 (0.03–4.06)
**Past trauma history**					
Nonrecidivist	1.00 Ref.	1.00 Ref.	1.00 Ref.	1.00 Ref.	1.00 Ref.
Single recidivist	1.33 (0.81–2.19)	1.22 (0.73–2.04)	1.43 (0.95–2.17)	1.45 (0.93–2.26)	1.94 (1.07–3.50)
Multirecidivist	2.88 (1.81–4.57)	2.69 (1.67–4.34)	2.27 (1.55–3.33)	2.31 (1.53–3.48)	2.52 (1.57–4.03)
**Substance detected**					
Alcohol	1.00 Ref.	1.00 Ref.	1.00 Ref.	1.00 Ref.	1.00 Ref.
Cannabis	0.74 (0.41–1.33)	0.71 (0.38–1.32)	0.69 (0.42–1.14)	0.71 (0.41–1.23)	0.53 (0.27–1.05)
Cocaine-amphetamine	1.23 (0.57–2.67)	1.35 (0.61–2.96)	0.90 (0.44–1.84)	1.09 (0.53–2.26)	1.08 (0.37–3.13)
Polydrugs	1.04 (0.69–1.55)	0.98 (0.64–1.50)	1.02 (0.73–1.41)	1.11 (0.77–1.59)	0.99 (0.61–1.60)

aHRR: Adjusted hazard rate ratio using Cox proportional hazards regression. aIRR: Adjusted incidence rate ratio using Poisson regression. ITT: Intention-to-treat. PP: Per-protocol. CACE: Complier average causal effect. NBI: Not offered brief intervention group. OBI: Offered brief intervention group. BIA: Brief intervention accepted group. Nonrecidivist: First-time trauma patients. Single recidivist: Patients with only one previous trauma. Multirecidivist: Patients with more than one previous trauma.

## Discussion

Our results strongly support the two hypotheses posed in the Introduction: trauma patients who tested positive for alcohol or illicit drug use had a higher rate of recidivism than those who tested negative, and among positive patients the recidivism rate was lower in those who received the BI. This latter result strongly supports the effect of BI in reducing the frequency of trauma recidivism in patients who screen positive for alcohol and/or other drug use. In the present study the more conservative (intention-to-treat) estimate yielded a 39% relative decrease, which rose to 52% decrease according to the CACE analysis. In addition, the first trauma after hospital discharge occurred 16 months earlier in the NBI group (who had less severe injuries at baseline) than in the OBI group. This emphasizes the benefit of the BI.

Given that in our setting almost half of trauma patients are admitted under the influence of alcohol and/or illicit drugs [38], the potential impact on public health of the implementation of SBIRT programs in trauma centers in Spain is enormous. In a cost-benefit analysis [39] with a similar estimated trauma risk reduction, screening and brief intervention for alcohol problems in trauma patients was found to be cost-effective (savings of USD3.81 for every USD1.00 spent), and the authors suggested that it should be routinely implemented. We note that the although the authors of that study only analyzed the impact of interventions on direct medical costs, we concur that the potential cost savings can be considered an additional advantage to the gains in other important indirect social benefits–which admittedly may be harder to quantify.

Our results are similar to those obtained in previous studies [27,28]; nevertheless, to our knowledge ours is the first long-term follow-up study (almost 5 years in some cases) of a patient cohort designed to measure the impact of BI on trauma recidivism. Previous efforts have focused on the decrease in alcohol and drug consumption as the primary outcome, and considered a reduction in recidivism as a secondary outcome [20,29–31]. It is important to take into account that the reduction in alcohol and illicit drug use resulting from BI may not be the only mediator between BI and the reduction in trauma recidivism: BI may also have a positive influence on other variables causally related to trauma, such as impulsive behavior [40–46] or trauma risk perception related to substance use. This perceived risk is particularly low in our social context, especially among consumers of substances other than alcohol, such as cannabis and cocaine [38]. According to the authors of the DRUID project (DRiving Under the Influence of Drugs) carried out in 18 European countries [47], the greater likelihood observed in some countries of detecting drivers under the influence of illicit drugs and medicines compared to drivers exposed to alcohol may be explained by the lower efforts and resources devoted to campaigns for accident prevention related to the consumption of these substances. These indirect mediators may explain why, although BI produces good short-term results in reducing alcohol consumption, these effects are diminished after 12 months [13,23], whereas the influence of the intervention in reducing the risk of trauma recidivism appears to be sustained in the long-term [27]. On the other hand, the effectiveness of screening and BI for drug use is being questioned by some studies [22,48]; however, our results show that the usefulness of these interventions goes beyond the reduction of consumption.

Although Gentilello et al. [27] used a health information system similar to ours to detect new traumas beyond 12 months, their design was sensitive only to injuries that resulted in hospitalization or death, whereas we were able to detect any trauma which received medical care regardless of whether it led to hospitalization.

A key element in our BI method was the addition of a booster phone call after 3 months in order to help patients to maintain the changes they resolved to make during hospitalization [26]. However, all previous studies also used a phone booster during the first month after hospital discharge [15,17,18,20,21]. We believe that a later booster session may help to enhance the effects of BI on recidivism by acting after physical recovery in most patients, i.e. when their motivation to abstain or limit substance use tends to decrease [26].

Regarding the other variables we investigated and their relation to recidivism, alcohol and illicit drug use and previous trauma history were the most important markers, confirming evidence from previous studies [3–7,49,50]. We tested the possibility that the effect of BI on the risk of recidivism might be modified by PHTx, but the interaction term between these two variables in the model was not significant. If the effectiveness of BI does not depend on a patient’s past trauma history, this variable may be useful to identify subgroups of high-risk patients for whom SBIRT programs should be prioritized [51,52], especially in situations when the lack of resources prevents the use of these programs for all patients who screen positive for alcohol or illicit drug use in the hospital emergency department.

We are aware that the main limitation of our study is the nonrandom assignment of our patients to the BI or no BI groups. Ethical reasons prevented this option, because when the MOTIVA project was implemented there was strong evidence supporting the effectiveness of SBIRT programs in reducing alcohol consumption. However, despite this evidence, it is noteworthy that this project is the only SBIRT-based initiative implemented in Spain thus far. In all cases, whether the patient received the BI or not was dependent only on the availability of an SBIRT interviewer, which in turn was related with the length of the patient’s hospital stay. A shorter stay may lead to less compliance with staff, which can lead to greater rejection of medical advice. Because variable “days of hospitalization”, along with the main baseline characteristics of the patients, was included in our multivariate models, we are confident that the adjusted association we found between BI and recidivism reflects a causal effect, although we cannot completely rule out alternative noncausal explanations.

Another possible drawback of our study is selection bias due to incomplete and differential follow-up. For example, patients may have been injured and received care in a different public health service area or may have switched to a private health insurance plan unconnected with the public health information network. We made an effort to complement follow-up through public health digital medical records with active follow-up by telephone. However, because of the high correlation between these two data sources and the low number of patients we detected as losses to follow-up, we are confident that this source of bias very likely had a low impact on our results.

## Conclusion

The results of this study suggest that a BI for hospitalized trauma patients who screened positive for alcohol and/or illicit drug use can halve the incidence of trauma recidivism. Although trauma recidivism in patients who received the brief motivational intervention was greater than in patients who screened negative for alcohol and/or illicit drug use on admission, the significant decrease compared to patients who screened positive and did not receive the intervention supports the need to implement screening and BI programs in trauma centers. Further research will be needed to explore how brief interventions influence factors other than the cessation of or reduction in alcohol and illicit drug use, such as impulsivity or trauma risk perception related to substance use, and to determine whether a positive effect on these factors might explain why decreased trauma recidivism appears to be maintained over time.

## Supporting information

S1 Data File (DTA)
